# Dysmobility Syndrome and Risk of Mortality for Community-Dwelling Middle-Aged and Older Adults: The Nexus of Aging and Body Composition

**DOI:** 10.1038/s41598-017-09366-z

**Published:** 2017-08-18

**Authors:** Wei-Ju Lee, Li-Kuo Liu, An-Chun Hwang, Li-Ning Peng, Ming-Hsien Lin, Liang-Kung Chen

**Affiliations:** 1 Aging and Health Research Center, National Yang Ming University, Taipei City, Taiwan; 2 Institute of Public Health, National Yang Ming University, Taipei City, Taiwan; 3 Department of Family Medicine, Taipei Veterans General Hospital Yuanshan Branch, Yilan County, Taiwan; 4 Center for Geriatrics and Gerontology, Taipei Veterans General Hospital, Taipei City, Taiwan

## Abstract

Dysmobility syndrome is a newly proposed concept to comprehensively consider bone-muscle-adiposity as a whole to associate with mortality and other adverse outcomes in the older adults. Little was known in Asian populations since the body composition was highly related to ethnicity. The study aimed to evaluate the association between dysmobility syndrome and mortality and to explore the most optimal operational definition for dysmobility syndrome. The prevalence of dysmobility syndrome was 3.9–10.1% based on different operational definitions of adiposity and skeletal muscle index. Subjects with dysmobility syndrome were older, more often to be women, having higher adiposity, lower lean body mass and bone mineral density. Multivariate Cox proportional hazard model showed that dysmobility and pre-dysmobility syndrome had higher risk of mortality than the robust group (Hazard ratio (HR): 11.3, 95% confidence interval (CI): 1.2–109.1; and HR 8.7, 95% CI 1.1-67.3, respectively). Overall, the modified operational definition of dysmobility syndrome in Asian populations using FNIH-adjusted skeletal muscle mass and waist circumference-defined adiposity may be the most optimal model for mortality prediction. Taking the nexus of body composition as a whole to evaluate the mortality risk of older adults is an important improvement beyond sarcopenia and osteoporosis.

## Introduction

Epidemiological studies have shown the interrelated associations between muscle strength, walking speed, sarcopenia, osteoporosis, body fat composition and mortality among older adults^1–5^. In current definitions of sarcopenia or skeletal muscle dysfunction, it needs both muscle quality and quantity to identify older people at risk for mobility limitation, falls and mortality^6–9^. Previous studies have disclosed that osteoporosis and sarcopenia eventually shared similar trends in the associations with adverse health outcomes among older adults^8, 10^. The interconnected relationship between bone and muscle with adverse outcomes led to the proposal of the bone-muscle unit as a whole to evaluate the effect of mobility to health^11^. Moreover, Binkley, *et al*., extended the concept from the bone-muscle unit to propose a new condition, i.e. dysmobility syndrome, which took the comprehensive consideration of bone, muscle and adiposity to early identify older people at risk^12^.

Operationally, dysmobility syndrome was defined by a score-based approach, which was similar to the definition of metabolic syndrome. Although the definition of dysmobility syndrome has been proposed, it remained to be a big challenge when the measurement of skeletal muscle was still under debate. Eventually, weight or height-adjusted skeletal muscle index identified people with very different clinical characteristics that weight-adjusted muscle index-defined low muscle mass tended to be overweight and obese while height-adjusted muscle index-defined low muscle mass tended to lean^13^. A recent study identified substantial differences in the prevalence of dysmobility syndrome and associated falls by using proposed definitions of skeletal muscle mass by the European Working Group for Sarcopenia in Older People (EWGSOP), the International Working Group on Sarcopenia (IWGS) and the Foundation for the National Institutes of Health Sarcopenia Project (FNIH)^14^. These differences may be even more significant in Asia due to higher adiposity of Asian people than Caucasians, especially in women^15^. The FNIH criteria proposed using body mass index (BMI) for the adjustment of skeletal muscle index to harmonize the definition of muscle index^7^, which may result in bigger discrepancy in Asian populations.

Dysmobility syndrome proposed the comprehensive approach of bone-muscle-adiposity to health of older people, and the association between dysmobility syndrome and adverse health outcomes has been established in some studies^12^. More research is needed to evaluate the impact of dysmobility syndrome on health of older people in different population with various characteristics. In particular, in Asia, the arbitrary score-based approach for definition of dysmobility syndrome deserves further investigation since the individual definition for adiposity and low muscle mass may differ from Western countries. Therefore, the main aim of this study intended to use a prospective population-based cohort to examine the association between dysmobility syndrome and mortality and to refine the operational definition of dysmobility syndrome through the outcome-based approach.

## Results

**Figure 1:**
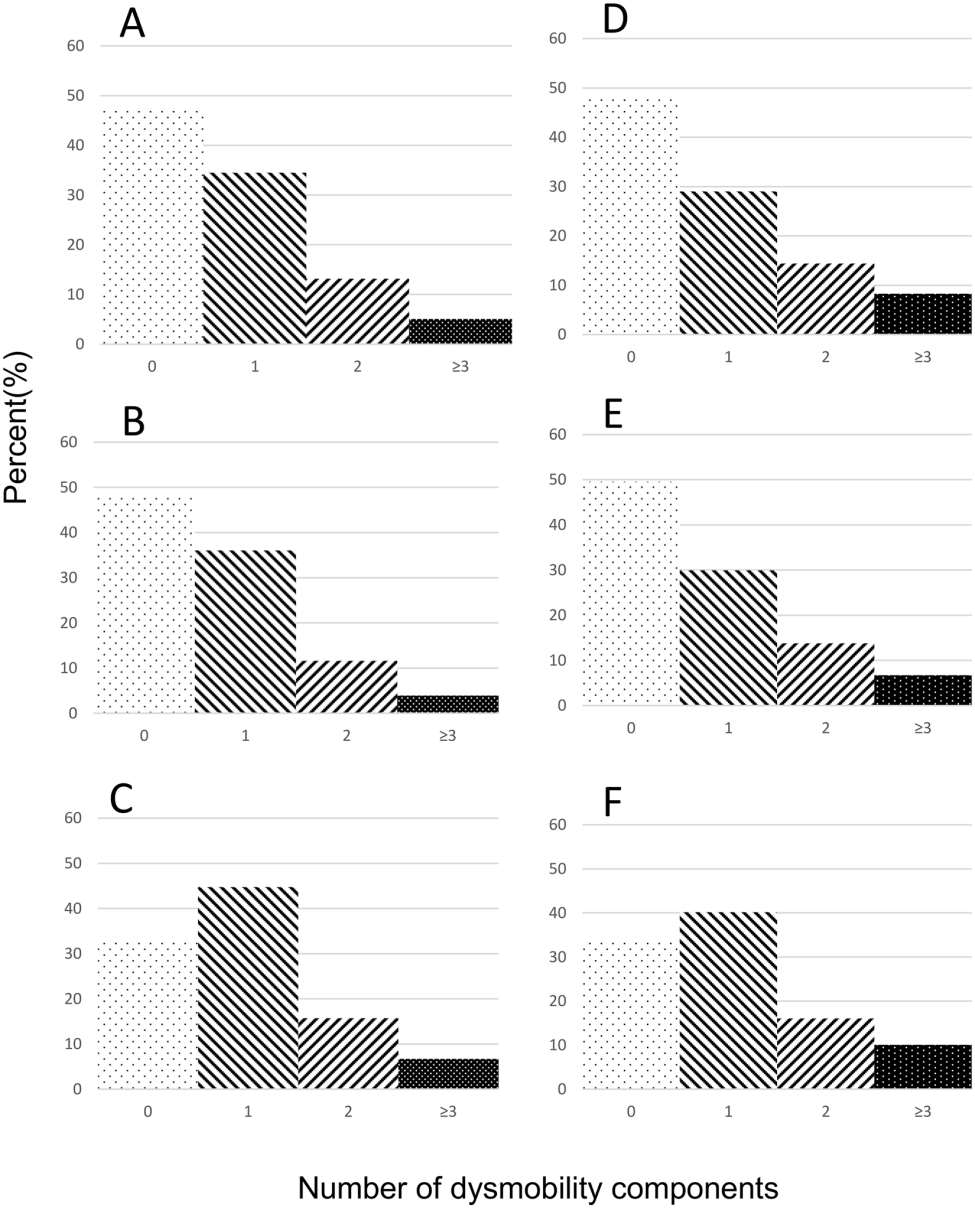
Distribution of dysmobility components by various muscle and fat definitions. Distribution of dysmobility syndrome by (**A**) original definition; (**B**) obesity determined by body mass index (**C**) obesity determined by central obesity (**D**) BMI adjusted muscle index (**E**) BMI adjusted muscle index plus obesity determined by BMI (**F**) BMI adjusted muscle index plus obesity determined by central obesity.

**Figure 2:**
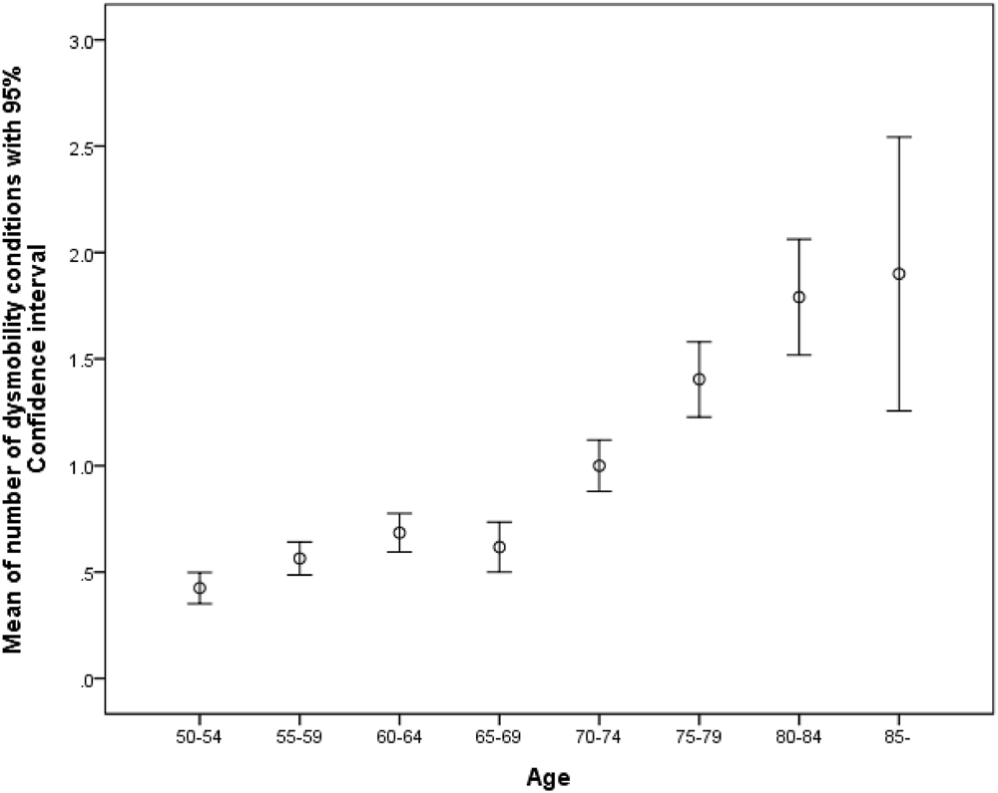
Mean of numbers of dysmobility conditions with 95% confidence interval versus age.

Table 1 summarized the characteristics of the whole study participants and compared differences by various status of dysmobility syndrome. In this study, the youngest participant was 50 years old and the oldest was 92. Among 89 (5.1%) participants with dysmobility syndrome, women were more predominant (6.5% versus 3.8%, *p* < 0.001). During the median follow-up of 2.6 years, 18 participants died (3.7 per 1000 person-years at risk). Among all determinants of dysmobility syndrome, distribution of dysmobility components were right skewed (Fig. 1). The number of components for dysmobility syndrome significantly increased with advancing age (*p* for trend < 0.001) (Fig. 2).

**Table 1:** Characteristics of participants of the I-Lan Longitudinal Aging Study.

	Total	Dysmobility status			
		Robust	Pre-dysmobility	Dysmobility	*p*
number	1757	831(47.3)	837(47.6)	89(5.1)	
Age(years)	63.8 ± 9.2	61.2 ± 8.1	65.2 ± 9.1	75.1 ± 8.5	**<0.001**
Men	825(46.9)	446(53.7)	348(41.6)	31(34.8)	**<0.001**
**Anthropometric measurements**					
Height(cm)	158.6 ± 8.0	160.3 ± 7.7	157.5 ± 7.8	152.5 ± 8.1	**<0.001**
Weight(Kg)	62.6 ± 11.0	62.1 ± 9.2	63.6 ± 12.3	58.6 ± 11.5	**<0.001**
Body mass Index(kg/m2)	24.8 ± 3.6	24.1 ± 2.5	25.6 ± 4.2	25.3 ± 5.0	**<0.001**
Central obesity	871(49.6)	301(36.2)	513(61.3)	57(64.0)	**<0.001**
**Health behavior**					
Smoke					**0.009**
never	1233(70.2)	568(68.4)	599(71.6)	66(74.2)	
current	307(17.5)	168(20.2)	132(15.8)	7(7.9)	
former	217(12.4)	95(11.4)	106(12.7)	16(18.0)	
Alcohol					**<0.001**
never	1036(59.0)	445(53.6)	527(63.0)	64(71.9)	
current	578(32.9)	326(39.2)	235(28.1)	17(19.1)	
former	143(8.1)	60(7.2)	75(9.0)	8(9.0)	
**Dual-energy X-ray absorptionmetry**					
Lean body mass(kg)	41.7 ± 8.2	43.6 ± 8.3	40.4 ± 7.7	35.8 ± 6.9	**<0.001**
Appendicular skeletal muscle(kg)	17.9 ± 4.1	19.1 ± 4.1	17.1 ± 3.8	14.7 ± 2.7	**<0.001**
Appendicular skeletal muscle/height^2^(kg/m^2^)	7.0 ± 1.1	7.3 ± 1.1	6.8 ± 1.1	6.2 ± 0.8	**<0.001**
Appendicular skeletal muscle/BMI(m^2^)	0.7 ± 0.2	0.8 ± 0.2	0.7 ± 0.1	0.6 ± 0.1	**<0.001**
Total fat mass(kg)	19.5 ± 7.0	17.2 ± 5.2	20.8 ± 7.9	21.7 ± 7.8	**<0.001**
Total fat percentage(%)	31.6 ± 8.7	28.4 ± 7.3	34.3 ± 8.9	35.5 ± 9.6	**<0.001**
Lumbar bone marrow density	1.0 ± 0.2	1.1 ± 0.2	1.0 ± 0.2	0.9 ± 0.2	**<0.001**
Hip bone marrow density	0.8 ± 0.1	0.9 ± 0.1	0.8 ± 0.1	0.7 ± 0.1	**<0.001**
**Physical performance**					
Walking speed(m/s)	1.5 ± 0.5	1.7 ± 0.5	1.4 ± 0.4	0.9 ± 0.4	**<0.001**
Handgrip strength(kg)	28.1 ± 9.5	31.8 ± 8.8	25.6 ± 8.8	17.2 ± 5.8	**<0.001**
**Function status**					
Fall	89(5.1)	0(0.0)	70(8.4)	19(21.4)	**<0.001**
The autonomy assessment scale	−0.2 ± 1.6	0.0 ± 0.3	−0.2 ± 1.5	−1.7 ± 5.4	**<0.001**
Charlson comorbidity index	1.0 ± 1.3	0.8 ± 1.1	1.1 ± 1.3	1.9 ± 1.5	**<0.001**

Numerical variables were expressed as mean ± standard deviation, categorized variables were expressed as number(%).

### Cox proportion hazard model for mortality prediction

Pre-dysmobility, dysmobility and low muscle strength were all significantly associated with mortality in age and sex adjusted and fully adjusted Cox regression analysis (Table 2). Table 2 also provided the estimated prevalence and hazard ratio for morality of each component of dysmobility syndrome. Adiposity was most common condition (28.9%), followed by low handgrip strength and osteoporosis. Among these conditions, only muscle strength and FNIH-defined sarcopenia (BMI-adjusted muscle index) were significantly associated with mortality.

**Table 2:** Prevalence and risk of mortality of dysmobility component conditions in the I-Lan Longitudinal Aging Study

Characteristic	n(%)	death(rate)		Age and sex adjusted		Full adjusted model	p
				HR(95% CI)	p	HR(95% CI)	
**Dysmobility**							
Robust	831(47.3)	1(0.1)		1		1	
Pre-dysmobility	837(47.6)	13(1.6)		8.5(1.1–65.8)	**0.040**	8.7(1.1–67.3)	**0.038**
Dysmobility	89(5.1)	4(4.5)		11.1(1.1–107.7)	**0.038**	11.3(1.2–109.1)	**0.037**
**Dysmobility components**							
High adiposity		Prevalence ranking					
Fat percentage	507(28.9)	6(1.2)		1.2(0.5–3.2)	0.703	1.4(0.5–3.8)	0.526
BMI	413(23.5)	2(0.5)		0.5(0.1–2.0)	0.299	0.5(0.1–2.2)	0.350
Central obesity	871(49.6)	12(1.4)	1	1.7(0.6–4.7)	0.297	2.0(0.7–5.6)	0.207
Low muscle mass							
ASM/height2	193(11.0)	3(1.6)		1.0(0.3–3.4)	0.9571	1.0(0.3–3.6)	0.983
ASM/BMI	330(18.8)	7(2.1)	2	1.5(0.6–3.9)	0.420	1.7(0.7–4.7)	0.265
Weak handgrip strength	297(16.9)	12(4.0)	3	5.1(1.7–14.9)	**0.003**	5.4(1.8–16.3)	**0.003**
Slow walking speed	57(3.2)	4(7.0)	6	2.1(0.6–7.1)	0.248	2.3(0.6–8.9)	0.212
Osteoporosis	209(11.9)	6(2.9)	4	2.0(0.7–5.8)	0.201	1.9(0.6–5.8)	0.285
Fall	89(5.1)	1(1.1)	5	0.7(0.1–5.3)	0.737	0.7(0.1–5.3)	0.705

ASM, appendicular skeletal muscle; BMI, body mass index; bold type indicated statistical significance; Full model was adjusted by age, sex, the autonomy assessment scale, Charlson comorbidity index, smoke and alcohol consumption.

### Discrimination between different definitions

**Figure 3:**
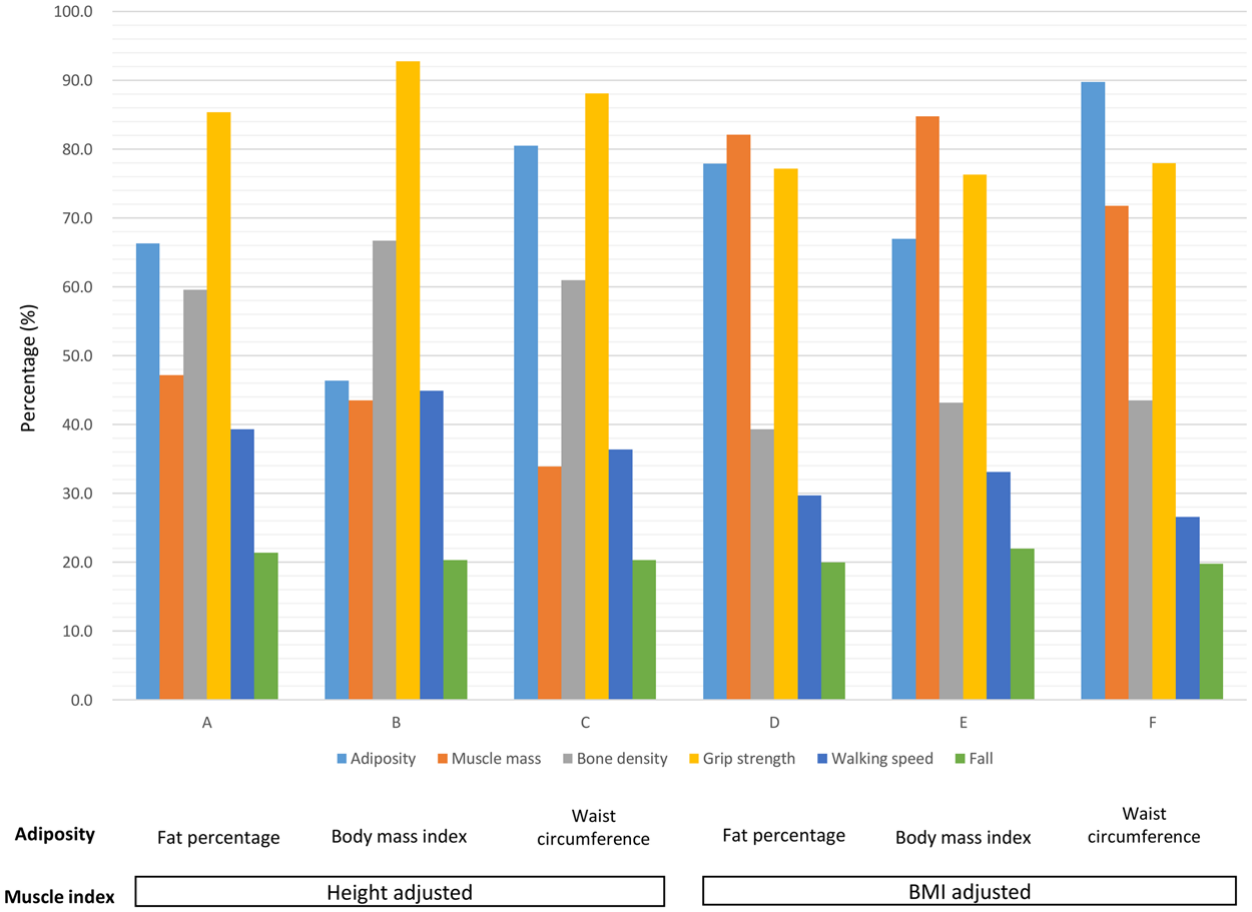
Distributions of dysmobility conditions by six different definitions of dysmobility syndrome.

Table 3 showed the effectiveness of mortality prediction of dysmobility syndrome by different definitions. In this study, we compared skeletal muscle index defined by different operational criteria. Both original dysmobility syndrome defined by Binkley, *et al*. and dysmobility syndrome using BMI-adjusted muscle index were associated with mortality. However, using the definition of dysmobility syndrome modified by BMI-adjusted muscle index eventually identified a higher prevalence of dysmobility syndrome. Major contributive components change and distribution of 6 components by different definitions were presented in Fig. 3.

**Table 3:** Prevalence and risk of mortality by various definitions of dysmobility syndrome.

Definition of dysmobility syndrome	Prevalence n(%)	Age and sex adjusted HR(95% CI)	Harrell’s R^2^	AIC	BIC
Original version (Binkley)			reference	224.3	227.9
Obesity as BMI ≥ 27 kg/m^2^					
Robust	851(48.4)	1	0.012	226.9	230.4
Pre-dysmobility	837(47.6)	4.7(1.0–21.0)			
Dysmobility	69(3.9)	4.6(0.7–30.8)			
Obesity as central obesity					
Robust	577(32.8)	1	0.017	228.1	231.6
Pre-dysmobility	1062(60.4)	4.2(0.5–33.2)			
Dysmobility	118(6.7)	8.3(0.9–80.1)			
BMI-adjusted muscle index and high body fat percentage					
Robust	849(48.3)	1	−0.005	223.2	226.8
Pre-dysmobility	763(43.4)	7.5(1.0–59.2)			
Dysmobility	145(8.3)	**14.0(1.6–123.1)**			
BMI-adjusted muscle index and obesity as BMI ≥ 27 kg/m^2^					
Robust	871(49.6)	1	0.013	227.2	230.8
Pre-dysmobility	768(43.7)	4.0(0.9–18.3)			
Dysmobility	118(6.7)	5.6(1.0–31.7)			
BMI-adjusted muscle index and obesity as central obesity					
Robust	592(33.7)	1	0.004	225.2	228.7
Pre-dysmobility	988(56.2)	3.3(0.4–26.8)			
Dysmobility	177(10.1)	**10.2(1.2–90.9)**			

HR, Hazard ratio; CI, confidence interval; AIC, Akaike Information Criterion; BIC, Bayesian information criterion; BMI, body mass index.

### Sensitivity analysis

A sensitivity analysis was conducted by excluding all participants died within one year after baseline interview. In the fully adjusted Cox proportional hazard model, subjects with dysmobility and pre-dysmobility syndrome had higher risk of mortality (HR 9.93 and 6.09; 95% CI 1.01–97.62 and 0.77–48.42, respectively) than robust ones. Moreover, FNIH-defined sarcopenia (HR 3.68, 95% CI 1.10–12.3) and low handgrip strength (HR:5.00, 95% CI 1.50–16.71) were both significantly associated with risk of mortality.

In the arbitrary score-based approach for definition of dysmobility syndrome, Table 4 summarized the comparison of prediction for mortality risk between different criteria other than 3 of 6 components and results showed the 3 of 6 components showed the better predictive power.

**Table 4:** Prevalence and mortality risk by different selected numbers of dysmobility components.

Dysmobility syndrome defined by different numbers of conditions	Prevalence n(%)	Age and sex adjusted HR(95% CI)	AIC	BIC
0 vs. 1 vs. ≧ 2				
Robust	831(47.3)	1	224.137	227.698
Pre-dysmobility	606(34.5)	7.7(0.9–62.6)		
Dysmobility	320(18.2)	**10.5(1.3–87.4)**		
0 vs. 1–2 vs. ≧ 3				
Robust	831(47.3)	1	224.301	227.863
Pre-dysmobility	837(47.6)	**8.5(1.1–65.8)**		
Dysmobility	89(5.1)	**11.1(1.1–107.7)**		
0 vs. 1–3 vs. ≧ 4				
Robust	831(47.3)	1	224.457	228.019
Pre-dysmobility	909(51.7)	**8.8(1.1–67.3)**		
Dysmobility	17(1.0)	10.6(0.6–183.7)		

AIC, Akaike Information Criterion; BIC, Bayesian information criterion.

Mortality risk of dysmobility syndrome was examined in the group aged 50–69 and the group aged 70 and over according to previous study.^16^ In the age and sex adjusted Cox proportional model, mortality risk for dysmobility were significant in the group aged 50–69 (HR 45.0, 95% 2.7–746.9) but the association was insignificant in the group aged 70 and over.

## Discussion

Dysmobility syndrome was significantly associated with the risk of mortality among middle-aged and older adults, and the results remained robust after excluding subjects who died within the first12 months of study. A dose-response effect between robust/predysmobility/dysmobility syndrome and mortality was observed. Moreover, using BMI-adjusted skeletal muscle index and waist circumference-defined obesity as the components for definition of dysmobility syndrome had highest AIC and BIC, which indicated better power for mortality prediction.

Due to the complexity of health in older people, researchers were keen to develop a comprehensive model to predict adverse outcomes of older people through a cluster of risk factors. In the clinical practice, sarcopenia, balance and other related factors were involved in the FRAX model to improve prediction for fragility fractures^17, 18^. Morley, *et al*., suggested to emphasize the mobility domains to sarcopenia as sarcopenia with limited mobility^19^. Binkley, *et al*., proposed the concept of dysmobility syndrome to capture sarcopenia, osteoporosis, mobility and balance simultaneously, which showed significant associations for the risk of falls, fractures, and even mortality of dysmobility syndrome^12^. Several cross-sectional studies have demonstrated the associations between previous fractures and dysmobility syndrome^14, 20^ and results from the current study supported that dysmobility syndrome significantly predicted mortality among middle-aged and older Taiwanese.

Hill, *et al*., indicated the need for refine the arbitrary cut-off points of dysmobility syndrome and suggested differences of anthropometric measures between Asian people and the Caucasian^21^. There were considerable debates about instruments for measurements of adiposity and muscle mass^7, 13, 22^, and results of this study suggested using waist circumference-defined adiposity and BMI-adjusted muscle index to define dysmobility syndrome in Asian populations. Among selection of muscle indices, FNIH-defined sarcopenia was significantly associated with mortality but height-adjusted muscle indices failed to reach statistical significance. Similar results were found in our previous studies that BMI-adjusted strength was more superior to handgrip strength *per se* in predicting cardiovascular risk^23^, Although adiposity ranked the highest prevalence among six components of dysmobility syndrome, muscle strength was the most powerful predictor for mortality, which was in consistent with the result from a national representative population-based study^5^, However, a study of 558 older men living in the retirement community showed that walking speed but not handgrip strength predicted all-cause mortality^24^, which may imply that handgrip strength may be a better mortality predictor among the otherwise healthy community-dwelling older adults. Nevertheless, dysmobility syndrome tried to capture adiposity-muscle-bone, strength, and performance in a score-based comprehensive approach,

Reported prevalence of dysmobility syndrome from Western countries was around 22–34%^12, 14, 16^, but a Korean study of 6,070 women with the mean age 74.1 years showed that only 43 subjects were positive for dysmobility syndrome^25^. Results from this current study showed that the prevalence of dysmobility syndrome ranged between 3.9–10.1% by using different operational definitions for muscle indices and adiposity. Prevalence of dysmobility syndrome by using BMI-adjusted muscle index (6.7–10.1%) were higher than that by using height-adjusted muscle index (3.9–6.7%). Those of dysmobility syndrome identified by BMI-adjusted muscle index were more likely to have higher adiposity and low muscle mass, and that defined by height-adjusted muscle index were more likely to be slowness or weakness. The prevalence of dysmobility syndrome in this study was similar to that from the National Health and Nutrition Examination Survey (NHANES) 1999–2002 if adiposity was determined by waist circumference. The risk of dysmobility syndrome has been reported higher in older adults aged 50–69 than those aged 70 and over^16^.

Using BMI-adjusted rather than height-adjusted muscle indices identify more individuals with dysmobility syndrome, who tend to have higher adiposity and lower muscle mass but less likely to be weakness and slowness. Among three significant predictive models of six different definitions, highest predictive ability for mortality was that with adiposity of waist circumferencebased and muscle mass of BMI-adjusted index. It is possible due to obesity-related health risk related to central distributed adiposity rather than total fat amount^26^ However, further investigations are needed to examine the effectiveness of the model for hip fracture prediction or other geriatric conditions.

Despite all efforts went into this study, there were still some limitations. First, in this study, history of falls was defined as previous fall within the past three months instead of last year in the original definition of dysmobility syndrome, which may underestimate the prevalence of dysmobility syndrome. Second, participants of the study cohort were living in rural region and otherwise healthy, which may also underestimate the impact of dysmobility syndrome on mortality. Third, sex-specific analysis was not done due to limited sizes of sample. However, the interaction between sex and dysmobility were insignificant. Nevertheless, this study not only described the epidemiology and association with mortality in Asian populations, but also clarify the most optimal modifications in the operational definitions of dysmobility syndrome.

## Conclusion

Dysmobility syndrome was significantly associated with mortality among community-dwelling middle-aged and older adults in Taiwan. Using waist circumference and BMI-adjusted muscle index were the most appropriate modified model for mortality prediction. Further intervention study is needed to evaluate the reversibility of dysmobility syndrome and mortality reduction.

## Methods

### Participants and study design

**Figure 4:**
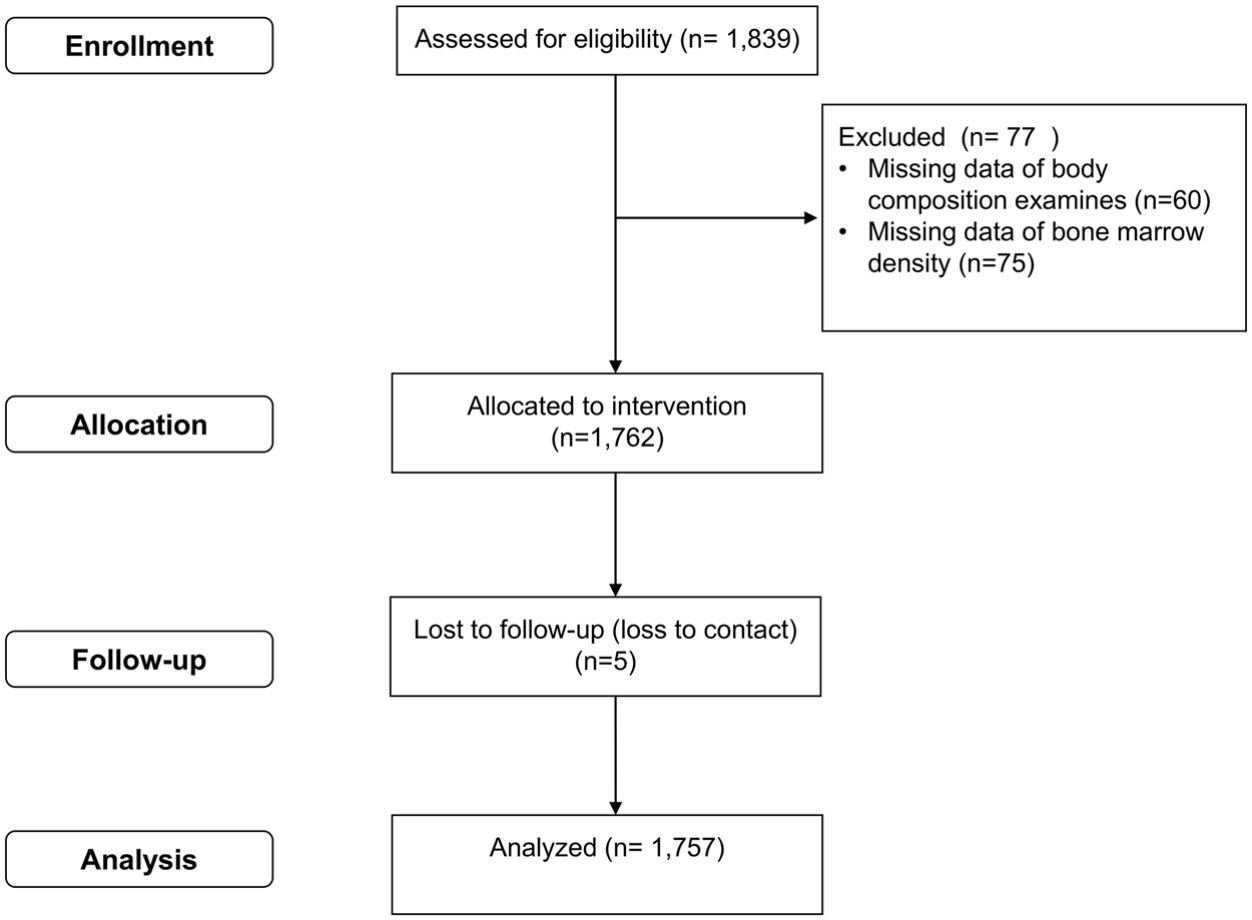
Participants derived from I-Lan Longitudinal Aging Study.

The I-Lan Longitudinal Aging Study(ILAS) was a prospective population-based cohort study, which aimed to investigate the association between sarcopenia, frailty and cognitive function of middle-aged and older adults in Taiwan. ILAS design, participant’s recruitment, and data collection have been reported elsewhere in detail^27^. Briefly, inhabitants aged 50 years and over in Yuanshan Township of I-Lan County in Taiwan were randomly selected from the household registrations of the county government and were invited through mail, postcard or telephone by research nurses. The inclusion criteria of ILAS were inhabitant aged 50 years of age or over living in Yuanshan Township presently and had no recent plan to move their residence. The exclusion criteria were (1) participants who could not communicate with research nurses, (2) those with limited life expectancy due to major illness (3) current residents in long-term facilities, and (4) those who were unable to complete evaluations due to poor function. Overall, 1,839 participants received face-to-face interviews by the research staff, and 1,779 of them received subsequent body composition tests and physical examinations. Among them, 77 participants were excluded for analysis due to data incompleteness. Survival status was documented and timed to the nearest month through telephone survey every three months until Jun 2015 and 5 participants were lost to follow-up. Overall, data of 1703 were obtained for analysis in this study (Fig. 4).

### Ethics statement and data availability

The observational design and reporting format follow STROBE (Strengthening the Reporting of Observational Studies in Epidemiology) guidelines^28^. A written informed consent was obtained from every participant. The institutional review board of the National Yang Ming University approved the study protocol. The design and procedures of the study were carried out in accordance with the principles of the Declaration of Helsinki. The datasets generated during and/or analysed during the current study are available from the corresponding author on reasonable request.

### Muscle strength and physical performance

In this study, muscle strength was measured by using handgrip strength (Smedlay’s Dynamo Meter; TTM, Tokyo, Japan). For every participant, the best measurement of three trials and allowed one pre-test trial at an upright standing position with straight down-side arms. The best performance was taken for analysis and those with muscle strength lower than 26 kg in men and 18 kg in women were referred as low muscle strength^9^. Gait speed were determined by a timed 6-meter walk and participants were instructed at usual paces with a static start without deceleration throughout a 6 m straight line at a more than 8 m length examine room. Those walked slower than 0.8 meter/second were defined as slow walking speed^8, 9^.

### Body composition and bone mineral density

Body fat mass, lean body mass and bone mineral density were measured by a whole body dual-energy X-ray absorptiometry (DXA) scan with a Lunar Prodigy instrument (GE Healthcare, Madison, WI, USA) in this study. Appendicular skeletal muscle mass (ASM) was calculated as the sum of the lean soft tissue mass of all four limbs. Height-adjusted muscle index and BMI-adjusted muscle index, calculated by appendicular skeletal muscle mass divided by height square (ASM/height^2^, kg/m^2^) and BMI (ASM/BMI, m^2^) were used to determined low muscle mass, respectively^7–9^. For all participants, height-adjusted skeletal muscle index lower than 7 kg/m^2^ and BMI-adjusted skeletal muscle mass index lower than 0.789 m^2^ in men were considered as low muscle mass; height-adjusted skeletal muscle mass lower than 5.4 kg/m^2^ and BMI-adjusted skeletal muscle mass < 0.512 m^2^ in women were referred as low muscle mass^7, 9^. Osteoporosis was determined by the diagnostic criteria from the World Health Organization (WHO) and those with T-score of lumbar or hip bone mineral density(BMD) less than −2.5 were defined as osteoporosis^29^.

### Dysmobility syndrome

Original definition of dysmobility syndrome was proposed by Binkley, *et al*.^12^, in which people had three or more of the following conditions were considered having dysmobility syndrome, i.e. high body fat, low muscle mass, osteoporosis, slow walking speed, weak muscle strength and fall in last three months. On the other hand, subjects with one or two conditions were categorized as pre-dysmobility in this study. Currently, six operative definitions of dysmobility syndrome had been reported by using different combinations of muscle index (height square adjusted and BMI adjusted) and various adiposity determinants (body percent fat > 30% in men and > 40% in women^12^, BMI ≥ 27 kg/m^2^ ^30^, waist circumference > 90 cm in men and > 80 cm in women^31^).

### Other confounders

Selected variables that possibly influenced vital status of participants in the multivariate statistical analysis and not included in the dysmobility syndrome were listed in this section. Tobacco consumption was categorized into three classes as current smoker, ex-smoker who quitted in the past 6 months, and non-smoker. Alcohol consumption was categorized into three groups as current drinker, ex-drinker who quitted in the past 6 months and non-drinker. The autonomy assessment scale (SMAF), a 29-items scale ranging from 0 to 87 points, was used to describe the general functional status, which measured activities of daily living (ADL), instrumental activities of daily living (IADL), mental function, and communications^32^. The Charlson’s Comorbidity Index (CCI), ranging from 1 to 6, was used describe the severity of underlying medical conditions^33^.

### Statistical analysis

In this study, numerical variables were expressed as mean ± standard deviation and categorical variables were expressed as proportions. Descriptive characteristics were compared by one-way ANOVA, chi-square analysis, or Fisher Exact test when appropriate. Cox proportional hazard regression model was used to explore the association between dysmobility status, individual component of dysmobility syndrome, sarcopenia and mortality. A test of assumption of proportionality indicated that no significant trend in hazards ratio with time (*p* = 0.794), which showed the assumption were not violated. Interaction between age, sex, SMAF, severity of disease, smoking, drinking and dysmobility syndrome were examined and showed no statistical significance. The mortality risk of dysmobility syndrome using different cutoff points of muscle index, walking speed, and adiposity compared to the results from the main analysis, which was conducted by comparison of Harrell’s R^2^, the Akaike Information Criterion (AIC) and the Bayesian information criterion (BIC)^34^. Harrell’sR^2^ estimates the proportion of explained variance in the proportional hazard model and is used to compare the performance in mortality prediction of dysmobility syndrome defined by different measures^35^. A secondary analysis was conducted to assess influence of the pre-existing illness on main results by excluding participants died within one year. In addition, impact of possible non-responder bias was examined by comparison between excluded and enrolled subjects. Although the excluded subjects were significantly older (66.9 versus 63.8 years), more likely to be current smokers (35.4% versus 17.5%) and more commonly to be men (63.1% versus 54.8%), however, they did not differ significantly from the 1757 participants in terms of gender, multimorbidity, drinking status, central obesity and mortality.

A two-sided *p*-value < 0.05 and 95% Confidence Intervals (CI) not spanning the null hypothesis values were considered statistically significant. All analyses were performed with the SAS statistical package, version 9.4 (SAS Institute, Inc., Cary, NC, USA).

## Electronic supplementary material

Dataset 1

## References

[1] Leong DP (2015). Prognostic value of grip strength: findings from the Prospective Urban Rural Epidemiology (PURE) study. Lancet.

[2] Studenski S (2011). Gait speed and survival in older adults. JAMA.

[3] Batsis JA, Mackenzie TA, Barre LK, Lopez-Jimenez F, Bartels SJ (2014). Sarcopenia, sarcopenic obesity and mortality in older adults: results from the National Health and Nutrition Examination Survey III. Eur J Clin Nutr.

[4] Landi F (2012). Sarcopenia and mortality among older nursing home residents. J Am Med Dir Assoc.

[5] Lee WJ, Peng LN, Chiou ST, Chen LK (2017). Physical Health Indicators Improve Prediction of Cardiovascular and All-cause Mortality among Middle-Aged and Older People: a National Population-based Study. Sci Rep.

[6] Cooper R, Bann D, Wloch EG, Adams JE, Kuh D (2015). “Skeletal muscle function deficit” in a nationally representative British birth cohort in early old age. J Gerontol A Biol Sci Med Sci.

[7] Studenski SA (2014). The FNIH sarcopenia project: rationale, study description, conference recommendations, and final estimates. J Gerontol A Biol Sci Med Sci.

[8] Cruz-Jentoft AJ (2010). Sarcopenia: European consensus on definition and diagnosis: Report of the European Working Group on Sarcopenia in Older People. Age Ageing.

[9] Chen LK (2014). Sarcopenia in Asia: consensus report of the asian working group for sarcopenia. J Am Med Dir Assoc.

[10] Weaver CM (2016). The National Osteoporosis Foundation’s position statement on peak bone mass development and lifestyle factors: a systematic review and implementation recommendations. Osteoporos Int.

[11] Tagliaferri C, Wittrant Y, Davicco MJ, Walrand S, Coxam V (2015). Muscle and bone, two interconnected tissues. Ageing Res Rev.

[12] Binkley N, Krueger D, Buehring B (2013). What’s in a name revisited: should osteoporosis and sarcopenia be considered components of “dysmobility syndrome?”. Osteoporos Int.

[13] Lee WJ (2013). Comparisons of sarcopenia defined by IWGS and EWGSOP criteria among older people: results from the I-Lan longitudinal aging study. J Am Med Dir Assoc.

[14] Clynes MA (2015). Definitions of Sarcopenia: Associations with Previous Falls and Fracture in a Population Sample. Calcif Tissue Int.

[15] Chen, L. K. *et al*. Recent Advances in Sarcopenia Research in Asia: 2016 Update From the Asian Working Group for Sarcopenia. *J Am Med Dir Assoc* (2016). 10.1016/j.jamda.2016.05.016 27372539

[16] Looker AC (2015). Dysmobility syndrome and mortality risk in US men and women age 50 years and older. Osteoporos Int.

[17] Lundin H (2017). Gait speed and one-leg standing time each add to the predictive ability of FRAX. Osteoporos Int.

[18] Yu R, Leung J, Woo J (2014). Sarcopenia combined with FRAX probabilities improves fracture risk prediction in older Chinese men. J Am Med Dir Assoc.

[19] Morley JE (2011). Sarcopenia with limited mobility: an international consensus. J Am Med Dir Assoc.

[20] Iolascon G, Moretti A, Giamattei MT, Migliaccio S, Gimigliano F (2015). Prevalent fragility fractures as risk factor for skeletal muscle function deficit and dysmobility syndrome in post-menopausal women. Aging Clin Exp Res.

[21] Hill KD, Farrier K, Russell M, Burton E (2017). Dysmobility syndrome: current perspectives. Clin Interv Aging.

[22] Schaap LA, Koster A, Visser M (2013). Adiposity, muscle mass, and muscle strength in relation to functional decline in older persons. Epidemiol Rev.

[23] Lee WJ, Peng LN, Chiou ST, Chen LK (2016). Relative Handgrip Strength Is a Simple Indicator of Cardiometabolic Risk among Middle-Aged and Older People: A Nationwide Population-Based Study in Taiwan. PLoS One.

[24] Chen PJ (2012). Predicting cause-specific mortality of older men living in the Veterans home by handgrip strength and walking speed: a 3-year, prospective cohort study in Taiwan. J Am Med Dir Assoc.

[25] Lim EJ, Noh JH (2015). Physical Function, Cognitive Function, and Depressive Symptoms in Elderly Women with Dysmobility Syndrome. Int J BioSci Bio Technol.

[26] Janssen I, Katzmarzyk PT, Ross R (2004). Waist circumference and not body mass index explains obesity-related health risk. Am J Clin Nutr.

[27] Liu LK (2014). Sarcopenia, and its association with cardiometabolic and functional characteristics in Taiwan: results from I-Lan Longitudinal Aging Study. Geriatr Gerontol Int.

[28] von Elm E (2007). The Strengthening the Reporting of Observational Studies in Epidemiology (STROBE) statement: guidelines for reporting observational studies. Prev Med.

[29] Kanis JA, Melton LJ, Christiansen C, Johnston CC, Khaltaev N (1994). The diagnosis of osteoporosis. J Bone Miner Res.

[30] Pan WH, Lee MS, Chuang SY, Lin YC, Fu ML (2008). Obesity pandemic, correlated factors and guidelines to define, screen and manage obesity in Taiwan. Obes Rev.

[31] O’Neill S, O’Driscoll L (2015). Metabolic syndrome: a closer look at the growing epidemic and its associated pathologies. Obes Rev.

[32] Hebert R, Carrier R, Bilodeau A (1988). The Functional Autonomy Measurement System (SMAF): description and validation of an instrument for the measurement of handicaps. Age Ageing.

[33] Charlson ME, Pompei P, Ales KL, MacKenzie CR (1987). A new method of classifying prognostic comorbidity in longitudinal studies: development and validation. J Chronic Dis.

[34] Vrieze SI (2012). Model selection and psychological theory: a discussion of the differences between the Akaike information criterion (AIC) and the Bayesian information criterion (BIC). Psychol Methods.

[35] Schemper M, Stare J (1996). Explained variation in survival analysis. Stat Med.

